# MMP-1 overexpression selectively alters inhibition in D1 spiny projection neurons in the mouse nucleus accumbens core

**DOI:** 10.1038/s41598-018-34551-z

**Published:** 2018-11-01

**Authors:** Nour Al-muhtasib, Patrick A. Forcelli, Katherine E. Conant, Stefano Vicini

**Affiliations:** 1 Georgetown University Medical Center, Department of Pharmacology & Physiology, Washington, 20007 D.C. USA; 2 Georgetown University Medical Center, Department of Neuroscience, Washington, 20007 D.C. USA; 3 Georgetown University Medical Center, Interdisciplinary Program in Neuroscience, Washington, 20007 D.C. USA

## Abstract

Protease activated receptor-1 (PAR-1) and its ligand, matrix metalloproteinase-1 (MMP-1), are altered in several neurodegenerative diseases. PAR-1/MMP-1 signaling impacts neuronal activity in various brain regions, but their role in regulating synaptic physiology in the ventral striatum, which is implicated in motor function, is unknown. The ventral striatum contains two populations of GABAergic spiny projection neurons, D1 and D2 SPNs, which differ with respect to both synaptic inputs and projection targets. To evaluate the role of MMP-1/PAR-1 signaling in the regulation of ventral striatal synaptic function, we performed whole-cell recordings (WCR) from D1 and D2 SPNs in control mice, mice that overexpress MMP-1 (MMP-1OE), and MMP-1OE mice lacking PAR-1 (MMP-1OE/PAR-1KO). WCRs from MMP1-OE mice revealed an increase in spontaneous inhibitory post-synaptic current (sIPSC), miniature IPSC, and miniature excitatory PSC frequency in D1 SPNs but not D2 SPNs. This alteration may be partially PAR-1 dependent, as it was not present in MMP-1OE/PAR-1KO mice. Morphological reconstruction of D1 SPNs revealed increased dendritic complexity in the MMP-1OE, but not MMP-1OE/PAR-1KO mice. Moreover, MMP-1OE mice exhibited blunted locomotor responses to amphetamine, a phenotype also observed in MMP-1OE/PAR-1KO mice. Our data suggest PAR-1 dependent and independent MMP-1 signaling may lead to alterations in striatal neuronal function.

## Introduction

Matrix metalloproteinase-1 (MMP-1) is a member of the MMP family of secreted, cell surface, zinc dependent endopeptidases^1^. While MMP-1 can process extracellular matrix molecules including collagens^2^, along with MMP-3 and -13 it is relatively unique in its ability to potently activate protease activated receptor-1 (PAR-1)^3^. Protease activated receptor-1 is a 7-transmembrane domain G-protein coupled cell surface receptor (GPCR) expressed on neurons, astrocytes, and microglia^4–7^. Proteolytic cleavage within the extracellular N-terminal domain of PAR-1 reveals a tethered ligand, which activates the receptor^8,9^. Endogenous activators of PAR-1 include thrombin and MMP-1^3,6^. PAR-1 activation is associated with multiple intracellular cascades, including increased release of calcium from intracellular stores, decreased cyclic AMP/protein kinase A signaling, and increased RhoA activity^10–12^. In addition, the receptor may be linked to β-arrestin dependent signaling cascades^9^.

The role of physiological PAR-1 in neuronal processing and health is complex. Studies have linked it to both neuronal protection and damage^13,14^, with divergent effects likely due to levels and localization of specific activators. Consistent with this, MMP levels must be “fine-tuned” in that too little or too much of a specific family member may be inimical to neuronal function^15^. This finely tuned balance may be altered in neurological disorders; for example, MMP-1 is highly expressed by activated astrocytes^16^ and its levels are increased in Alzheimer’s disease. Similarly, PAR-1 expression is increased in astrocytes in Parkinson’s disease and HIV encephalitis^17,18^. Though beyond scope of the present study, PAR-1 activation can also contribute to endothelial injury, as well as tumoral, ischemic and inflammatory pathology^19^.

Despite abundant expression of PAR-1 and activating proteases in varied brain regions, few studies have examined the effects of this axis on neurotransmission in the striatum, in particular the nucleus accumbens core. Previously published work has shown that MMP activity and/or PAR-1 activation can alter neuronal excitation and inhibition, promote NMDA receptor function, and induce long term potentiation in the hippocampus^20–24^. Recent work also demonstrated that MMP dependent PAR activation is critical to increased NMDA function in the hippocampal stratum radiatum^21^.

PAR-1 and it activators are also expressed in the striatum^25^. The role of the MMP-1/PAR-1 axis in regulating striatal neurophysiology, however, has not been yet examined. PAR activating MMPs are increased in a murine model of Parkinson’s disease (PD)^26^, and PD symptomatology is exacerbated by alterations of synaptic transmission onto D1 and D2 SPNs and alterations of their excitability^27,28^. One possibility is that MMP-1 dependent PAR-1 activation leads to an imbalance on synaptic transmission onto D1 and D2 SPNs. In the present study we test this hypothesis with specific tools including whole-cell recordings and MMP-1 transgenic mice in which specific striatal neurons are fluorescently labeled.

## Results

### Alterations in inhibitory and excitatory transmission

We compared the average frequency, amplitude, rise time, and decay time of synaptic currents in D1 and D2 SPNs in control^29^, MMP-1OE mice (mice that overexpress MMP-1), and MMP-1OE/PAR-1KO mice (MMP-1OE mice that lack PAR-1). Both spontaneous inhibitory postsynaptic currents (sIPSCs) and miniature IPSCs (mIPSCs) as well as miniature excitatory PSCs (mEPSCs) were measured to probe the source of alterations in inhibitory and excitatory currents.

**Figure 1:**
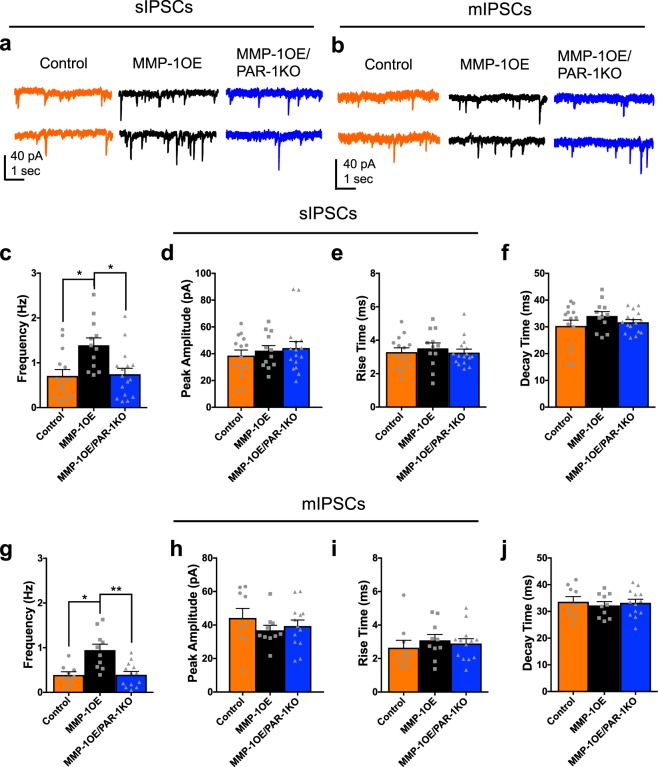
Increased sIPSC and mIPSC frequency in D1 SPNs of MMP-1OE mice is not seen in MMP-1OE/PAR-1KO mice. Representative traces of (**a**) sIPSC and (**b**) **mIPSC** whole-cell voltage-clamp recordings from D1 SPNs in a control mouse (left), MMP-1OE mouse (middle), and MMP-1OE/PARKO mouse (right). (**c**) sIPSC frequency in D1 SPNs in MMP-1OE mice is increased compared to control mice (p = 0.0165) and MMP-1OE/PAR-1KO mice (p = 0.0219). Comparison of sIPSC peak amplitude (**d**), rise time (**e**), and decay time (**f**) between control, MMP-1OE, and MMP-1OE/PAR-1KO mice. N = control (14 cells from 10 animals), MMP-1OE (12 cells from 10 animals), and MMP-1OE/PAR-1KO (16 cells from 11 animals). Kruskal-Wallis test with Dunn’s multiple comparisons. (**g**) mIPSC frequency in D1 SPNs in MMP-1OE mice is increased compared to D1 SPNs of control mice (*p = 0.0102) and MMP-1OE/PAR-1KO mice (**p = 0.0067). Comparison of sIPSC peak amplitude (**h**), rise time (**i**), and decay time (**j**) between control, MMP-1OE, and MMP-1OE/PAR-1KO mice. The data source for controls is the same as in Al-muhtasib *et al*., 2018^29^. N = control (9 cells from 8 animals), MMP-1OE (10 cells from 9 animals), and MMP-1OE/PAR-1KO (13 cells from 11 animals). Kruskal-Wallis test with Dunn’s multiple comparisons.

sIPSC frequency was significantly increased in D1 SPNs from MMP-1OE mice (1.4 ± 0.16 Hz) in comparison to those from either control^29^ (0.71 ± 0.14 Hz, p = 0.0165) or MMP-1OE/PAR-1KO mice (0.74 ± 0.13 Hz, p = 0.0219, Fig. 1a,c). sIPSC frequency in D1 SPNs did not differ between control and MMP-1OE/PAR-1KO mice (p > 0.9999). sIPSC peak amplitude (p = 0.8827), rise time (p = 0.5522), and decay time (0.4744) from D1 SPNs did not differ as a function of genotype (Fig. 1d,e,f).

To determine whether the alterations in inhibitory transmission are due to action potential dependent or independent release of neurotransmitter we also assed tetrodotoxin -insensitive mIPSCs (TTX, voltage-gated sodium channel blocker). mIPSC frequency was increased in D1 SPNs of MMP-1OE (0.95 ± 0.13 Hz) mice in comparison those from either control^29^ (0.39 ± 0.08 Hz, p = 0.0102) or MMP-1OE/PAR-1KO mice (0.39 ± 0.07 Hz, p = 0.0067, Fig. 1b,g). mIPSC peak amplitude (p = 0.5378), rise time (p = 0.5019), and decay time (p = 0.7770) did not differ as a function of genotype (Fig. 1h,i,j).

**Figure 2:**
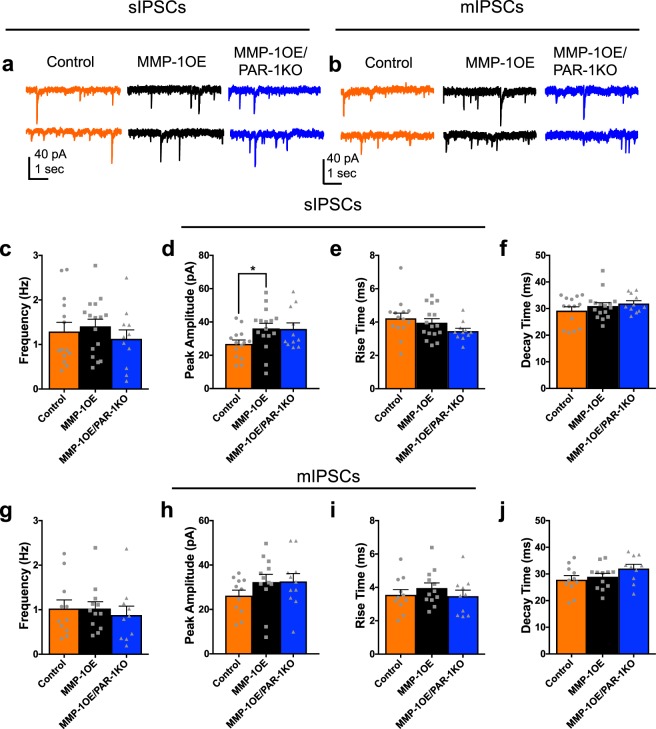
sIPSC and mIPSC frequency in D2 SPNs did not differ as a function of genotype. Representative traces of **(a)** sIPSC and **(b)** mIPSC whole-cell voltage-clamp recordings from D2 SPNs in a control mouse (left), MMP-1OE mouse (middle), and MMP-1OE/PARKO mouse (right). (**c**) sIPSC frequency in D2 SPN did not differ as a function of genotype (p = 0.4931). (**d**) sIPSC peak amplitude in D2 SPNS did not differ between MMP-1OE/PARKO (35.8 ± 3.64 pA) and either control (26.8 ± 2.33 pA, p = 0.3733) or MMP-1OE (36.1 ± 3.14 pA, p > 0.9999) mice. However, sIPSC peak amplitude was significantly smaller in control mice compared to MMP-1 OE mice (p = 0.0456). Rise time (p = 0.0.0974) (**e**) and decay time (p = 0.6770) (**f**) did not differ between control, MMP-1OE, and MMP-1OE/PAR-1KO mice. N = control (14 cells from 11 animals), MMP-1OE (16 cells from 11 animals), and MMP-1OE/PAR-1KO (11 cells from 10 animals). Kruskal-Wallis test with Dunn’s multiple comparisons. (**g**) mIPSC frequency in D2 SPN did not differ as a function of genotype (p = 0.6489). Comparison of mIPSC peak amplitude (**h**), rise time (**i**), and decay time (**j**) between control, MMP-1OE, and MMP-1OE/PAR-1KO mice. The data source for controls is the same as in Al-muhtasib *et al*., 2018^29^. N = control (11 cells from 7 animals), MMP-1OE (12 cells from 10 animals), and MMP-1OE/PAR-1KO (10 cells from 9 animals). Kruskal-Wallis test with Dunn’s multiple comparisons.

Conversely, sIPSC frequency in D2 SPNs was comparable between control^29^ (1.3 ± 0.21 Hz), MMP-1OE (1.4 ± 0.16 Hz) and MMP-1OE/PAR-1KO (1.1 ± 0.20 Hz) mice (p = 0.4931, Fig. 2a,c). sIPSC peak amplitude in D2 SPNS did not differ between MMP-1OE/PARKO (35.8 ± 3.64 pA) and either control^29^ (26.8 ± 2.33 pA, p = 0.3733) or MMP-1OE (36.1 ± 3.14 pA, p > 0.9999, 2d) mice. However, sIPSC peak amplitude was significantly smaller in control mice compared to MMP-1 OE mice (p = 0.0456). sIPSC rise time (p = 0.0974) and decay time (p = 0.6770) did not differ as a function of genotype (Fig. 2e,f).

Additionally, mIPSC frequency in D2 SPNs did not differ between control^29^ (1.0 ± 0.19 Hz), MMP-1OE (1.0 ± 0.15 Hz), and MMP-1OE/PAR-1KO (0.88 ± 0.20 Hz) mice (p = 0.6489, Fig. 2b,g). mIPSC average amplitude (p = 0.2707), rise time (p = 0.4866), and decay time (p = 0.1268) did not differ as a function of genotype (Fig. 2h,i,j).

**Figure 3:**
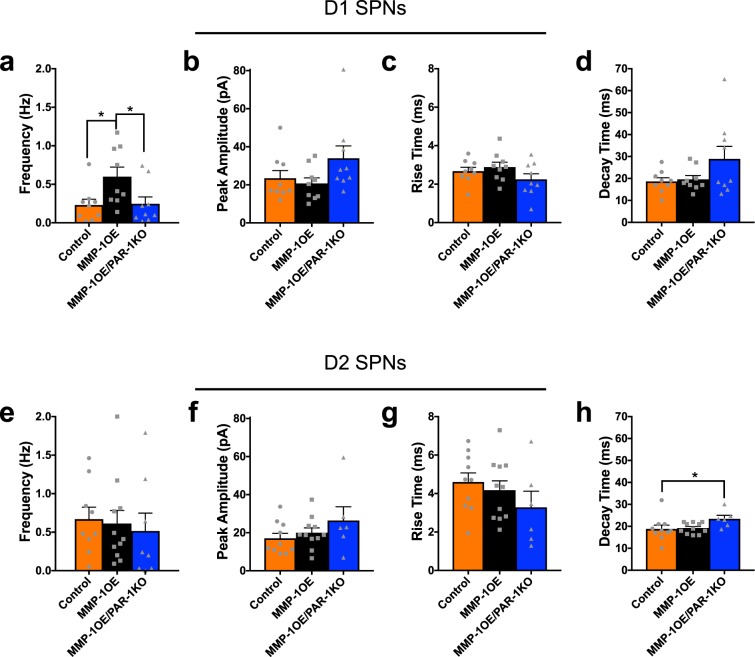
Increased mEPSC frequency in D1 SPNs of MMP-1OE mice is not seen in MMP-1OE/PAR-1KO mice. (**a**) mEPSC frequency was increased in D1 SPNs of MMP-1OE mice (0.60 ± 0.12 Hz) compared to control (0.23 ± 0.08 Hz, p = 0.0427) and MMP-1OE PAR-1KO mice (0.25 ± 0.09 Hz, p = 0.0256). Comparison of mIPSC peak amplitude (**b**), rise time (**c**), and decay time (**d**) in D1 SPNs between control, MMP-1OE, and MMP-1OE/PAR-1KO mice. N = control (9 cells), MMP-1OE (9 cells), and MMP-1OE/PAR-1KO (9 cells). Kruskal-Wallis test with Dunn’s multiple comparisons. (**e**) mEPSC frequency in D2 SPN did not differ as a function of genotype. Comparison of mIPSC peak amplitude (**f**), rise time (**g**), and decay time (**h**) in D2 SPNs between control, MMP-1OE, and MMP-1OE/PAR-1KO mice. The data source for controls is the same as in Al-muhtasib *et al*., 2018^29^. N = control (9 cells), MMP-1OE (11 cells), and MMP-1OE/PAR-1KO (6 cells). Kruskal-Wallis test with Dunn’s multiple comparisons.

Excitatory transmission was also assessed during biccuculline methobromide (BMR, GABA_A_ receptor antagonist) application to block the occurrence of GABAergic IPSCs. mEPSC frequency was increased in D1 SPNs of MMP-1OE mice (0.60 ± 0.12 Hz) compared to control^29^ (0.23 ± 0.08 Hz, p = 0.0427) and MMP-1OE PAR-1KO mice (0.25 ± 0.09 Hz, p = 0.0256, 3a). Peak amplitude (p = 0.1130), rise times (p = 0.3327), and decay times (p = 0.4357) for mEPSCs in D1 SPNs did not differ as a function of genotype (Fig. 3b,c,d). mEPSC frequency (p = 0.4236), peak amplitude (p = 0.5511) and rise times (p = 0.3142) in D2 SPNs did not differ as a function of genotype (Fig. 3e,f,g).

mEPSC decay time in D2 SPNS did not differ between MMP-1OE (19.2 ± 0.74 pA) and either control^29^ (18.8 ± 1.77 pA, p > 0.9999) or MMP-1OE/PAR-1KO (23.4 ± 1.59 pA, p = 0.1488, 3 h) mice. However, mEPSC decay time was significantly smaller in control mice compared to MMP-1 OE/PAR-1KO mice (p = 0.0497).

### Passive and active properties

We assessed the passive and active membrane properties of D1 and D2 SPNs in the control, MMP-1OE, MMP-1OE/PARKO mice. Membrane properties for D1 and D2 SPNs are compared and summarized in Table 1. The membrane resistance of D1 SPNs of the MMP-1OE/PAR-1KO (258 ± 31 MΩ) mice was increased compared to control^29^ (144 ± 11 MΩ, p = 0.0128) and MMP-1OE (141 ± 10 MΩ, p = 0.0026) mice. The membrane resistance of D2 SPNs did not differ between the three genotypes (p = 0.8143). Additionally, the time constant of D1 SPNs in the MMP-1OE/PAR-1KO mice (12 ± 1.3 ms) was increased compared to control^29^ (7 ± 0.8 ms, p = 0.0026) and MMP-1OE (8 ± 0.8 ms, p = 0.0224) mice, but the time constant of D2 SPNs did not differ as a function of genotype (p = 0.2648). Neither membrane capacitance (D1 SPNs p = 0.6951, D2 SPNs p = 0.6588) or resting membrane potential (D1 SPNs p = 0.2978, D2 SPNs p = 0.2109) differed as function of genotype for either D1 or D2 SPNs. We also quantified the effect of BMR on D1 SPNs of control and MMP-1OE mice and failed to see a significant difference in tonic current (data not shown).

**Table 1:** Summary of passive properties of D1 and D2 SPNs as a function of genotype. The membrane resistance of D1 SPNs of the MMP-1OE/PAR-1KO mice was increased compared to control (p = 0.0128) and MMP-1OE mice (p = 0.0026).

	D1 SPNs			D2 SPNs		
	Control	MMP-1OE	MMP-1OE/ PAR-1KO	Control	MMP-1OE	MMP-1OE/ PAR-1KO
Membrane Resistance (MΩ)	144 ± 11	141 ± 10	258 ± 31*	203 ± 32	236 ± 29	230 ± 32
Time Constant (ms)	7.0 ± 0.8	8.1 ± 0.8	12 0.2 ± 1.3*	8.5 ± 1.2	11 ± 1.4	11 ± 1.4
Capacitance (pF)	55 ± 6.4	57 ± 3.9	48 ± 5.2	45 ± 4.6	51 ± 6.5	53 ± 6.4
RMP (mV)	−69 ± 1.95	−71 ± 1.0	−71 ± 1.4	−66 ± 2.1	−71 ± 2.3	−70 ± 2.1
# of cells	19	27	15	17	16	15
# of animals	13	20	12	13	12	11

The time constant of D1 SPNs in the MMP-1OE/PAR-1KO mice was increased compared to control (p = 0.0026) and MMP-1OE (p = 0.0224). The data source for controls is the same as in Al-muhtasib *et al.*, 2018^29^.

### Altered excitability and dendritic complexity

**Figure 4:**
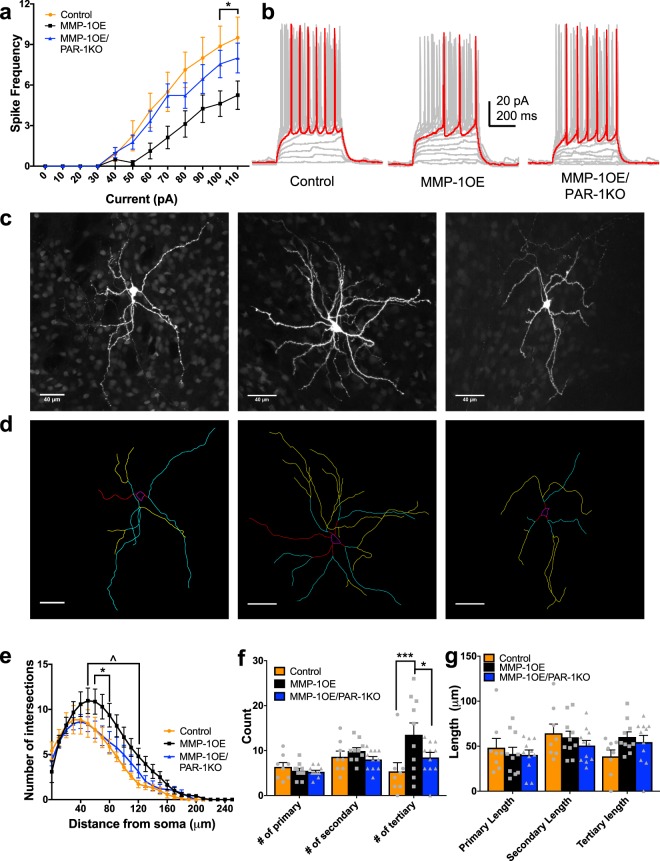
Altered D1 SPN excitability and morphology in MMP-1OE mice. (**a**) MMP-1OE D1 SPNs display decreased excitability compared to control and MMP-1OE/PAR-1KO D1 SPNs. Increasing current injections led to increased action potential numbers (F_11,242_ = 75.29, p < 0.0001). There was no main effect of genotype (F_2,22_ = 3.044, p = 0.0681), but there was an interaction between these variables (F_22,242_ = 2.588, p = 0.0002). (**b**) Representative traces from current clamp recordings of D1 SPNs from control (left), MMP-1OE (middle), and MMP-1OE/PAR-1KO (right) mice. D1 SPNs N = control (8 cells from 7 animals), MMP-1OE (8 cells from 6 animals), and MMP-1OE/PAR-1KO (9 cells from 7 animals). 2-way ANOVA. (**c**) Z projections of confocal images stacks of biocytin filled D1 SPNs. Slices are from control (left), MMP-1OE (middle), and MMP-1OE/PAR-1KO (right). (**d**) Neurons from **c** traced. Soma is in magenta, primary dendrites are in red, secondary in cyan, and tertiary in yellow. Images from control (left), MMP-1OE (middle), and MMP-1OE/PAR-1KO (right). (**e**) MMP-1OE D1 SPNs have increased dendritic complexity in comparison to control and MMP-1OE/PAR-1KO mice between (*) 60 μm (p_control_ = 0.0196, p_MMP-1OE/PAR-1KO_ = 0.0099) and 80 μm (p_control_ = 0.0070, p_MMP-1OE/PAR-1KO_ = 0.0137). Additionally, D1 SPNS in MMP-1OE mice displayed increased dendritic complexity compared to controls (^) between 90 μm (p = 0.0085) and 120 μm (p = 0.0209). There was an effect of distance from the soma (F_25,650_ = 84.17, p < 0.0001). There was no main effect of genotype (F_2,26_ = 2.533, p = 0.0989), but there was a significant interaction between these variables (F_50,650_ = 1.576, p = 0.0083). (**f**) MMP-1OE D1 SPNs exhibit increased number of tertiary branches in comparison to control (***p = 0.0002) and MMP-1OE/PAR-1KO (*p = 0.0152) mice. Number of branches increased as a function of branch complexity (F_2,52_ = 8.121, p = 0.0009). There was no main effect of genotype (F_2,26_ = 2.764, p = 0.0816), but there was an interaction between these two variables (F_4,52_ = 8.121, p = 0.0068). (**g**) Branch length did not differ between the three genotypes. There was an effect of branch complexity on length (F_2,52_ = 4.087, p = 0.0225), but no main effect of genotype (F_2,26_ = 0.2904, p = 0.7504) or an interaction between these variables (F_4,52_ = 1.896, p = 0.1251).The data source for controls is the same as in Al-muhtasib et al., 2018^29^. N = control (8 cells from 8 animals), MMP-1OE (10 cells from 9 animals), and MMP-1OE/PAR-1KO (11 cells from 10 animals). 2-way ANOVA and Kruskal-Wallis test with Dunn’s multiple comparisons test.

Our data revealed differences in excitability as a function of genotype with correlating alterations in dendritic complexity. Increasing depolarizing current injections led to an increase of action potentials fired in D1 SPNs (F_11,242_ = 75.29, p < 0.0001). There was no main effect of genotype (F_2,22_ = 3.044, p = 0.0681), but there was significant interaction between these variables (F_22,242_ = 2.588, p = 0.0002). The source of this difference was decreased D1 SPN excitability in the MMP-1OE mice compared to control and MMP-1OE/PAR-1KO mice at 100 pA injections (p_control_ = 0.0013, p_MMP-1OE/PAR-1KO_ = 0.0321) and 110 pA injections (p_control_ = 0.0013, p_MMP-1OE/PAR-1KO_ = 0.0480, Fig. 4a,b).

D1 SPNs of MMP-1OE mice displayed increased dendritic complexity as compared to control and MMP-1OE/PAR-1KO mice as measured by Sholl analysis (Fig. 4c–e). As expected, dendritic complexity increased as a distance from the soma (F_25,650_ = 84.17, p < 0.0001). There was no main effect of genotype (F_2,26_ = 2.533, p = 0.0989), but there was a significant interaction between these variables (F_50,650_ = 1.576, p = 0.0083). D1 SPNs of MMP-1OE mice displayed increased dendritic complexity in comparison to controls and MMP-1OE/PAR-1KO mice between 60 μm (p_control_ = 0.0196, p_MMP-1OE/PAR-1KO_ = 0.0099) and 80 μm (p_control_ = 0.0070, p_MMP-1OE/PAR-1KO_ = 0.0137). Additionally, D1 SPNS in MMP-1OE mice displayed increased dendritic complexity compared to controls between 90 μm (p = 0.0085) and 120 μm (p = 0.0209). The altered dendritic complexity was also observed as an increased number of tertiary branches of D1 SPNs in MMP-1OE mice compared to control (p = 0.0002), and MMP-1OE/PAR-1KO (p = 0.0152) mice (Fig. 4f). The number of branches increased as a function of branch complexity (F_2,52_ = 8.121, p = 0.0009). There was no main effect of genotype (F_2,26_ = 2.764, p = 0.0816), but there was an interaction between these two variables (F_4,52_ = 8.121, p = 0.0068). Branch length did not differ across the three genotypes (Fig. 4g). There was an effect of branch complexity on length (F_2,52_ = 4.087, p = 0.0225), but no main effect of genotype (F_2,26_ = 0.2904, p = 0.7504) or an interaction between these variables (F_4,52_ = 1.896, p = 0.1251).

**Figure 5:**
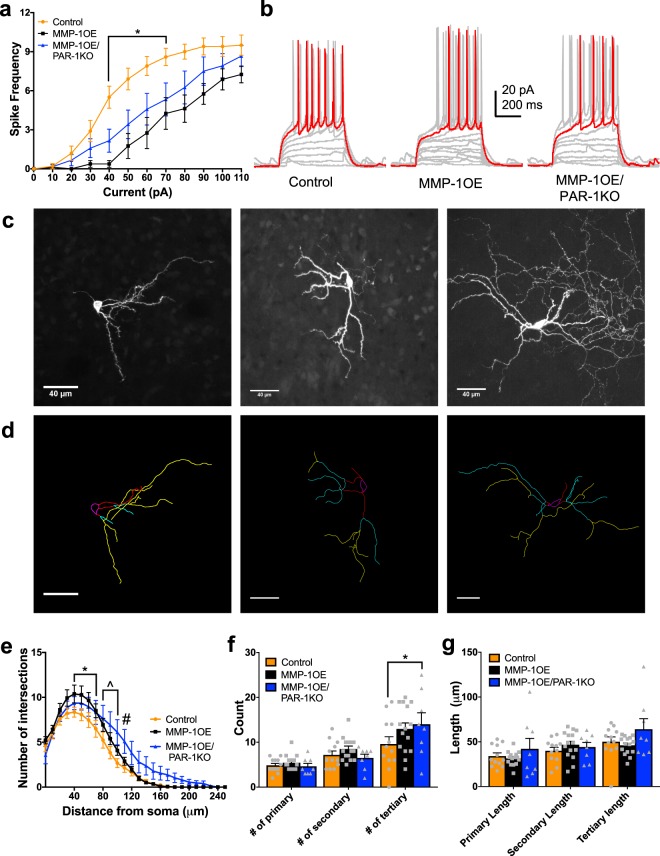
Altered D2 SPN excitability and morphology in MMP-1OE and MMP-1OE/PAR-1KO mice. (**a**) Input/output curve for D2 SPNs displaying decreased excitability in MMP-1OE and MMP-1OE/PAR-1KO mice. Increasing amplitude of depolarizing current steps led to a significant increase in action potential firing rate in D2 SPNs for all genotypes (F_11,297_ = 101.6, p < 0.0001). There was a main effect of genotype (F_2,27_ = 5.128, p = 0.0129), and a significant interaction between genotype and current injection (F_22,297_ = 3.152, p = < 0.0001). (**b**) Representative current clamp recordings of D2 SPNs from control (left), MMP-1OE (middle), and MMP-1OE/PAR-1KO (right). N = control (10 cells from 6 animals), MMP-1OE (8 cells from 6 animals), and MMP-1OE/PAR-1KO (12 cells from 11 animals). (**c**) Z stack projections of confocal images of biocytin filled D2 SPNs. Slices from control (left), MMP-1OE (middle), and MMP-1OE/PAR-1KO (right) mice. (**d**) Neurons from **c** traced. Soma is in magenta, primary dendrites in red, secondary in cyan, and tertiary in yellow. Images from control (left), MMP-1OE (middle), and MMP-1OE/PAR-1KO (right). (**e**) Control D2 SPNs displayed decreased complexity between 40 μm (p = 0.0263) and 70 μm (p = 0.0287) compared to MMP-1OE mice (*). MMP-1OE/PAR-1KO mice displayed increased complexity compared to control mice (^) between 80 μm (p = 0.0259) and 100 μm (p = 0.0006). At 110 μm, MMP-1OE/PAR-1KO mice displayed increased complexity compared to control (p = 0.0039) and MMP-1OE mice (p = 0.0329, #). There was increased complexity as a function of distance from the soma (F_25,800_ = 143.5, p < 0.0001), no main effect of genotype (F_2,32_ = 2.59, p = 0.0.0906), but there was a significant interaction between these variables (F_50,800_ = 1.972, p = 0.0001). (**f**) Control D2 SPNs exhibit decreased number of tertiary branches compared to MMP-1OE MMP-1OE/PAR-1KO mice (*p = 0.0369). There was a main effect of branching (F_2,64_ = 30.26, p < 0.0001), but no main effect of genotype (F_2,32_ = 1.962, p = 0.1571) or an interaction of these variables (F_4,64_ = 1.384, p = 0.2496). (**g**) Branch length did not differ across the genotypes. There was an effect of branching (F_2,64_ = 10.01, p = 0.0002), but no effect of genotype (F_2,32_ = 1.617, p = 0.2143), or an interaction of these variables (F_4,64_ = 1.216, p = 0.3128). The data source for controls is the same as in Al-muhtasib et al., 2018^29^. N = control (12 cells from 8 animals), MMP-1OE (15 cells from 8 animals), and MMP-1OE/PAR-1KO (8 cells from 8 animals). 2-way ANOVA and Kruskal-Wallis test with Dunn’s multiple comparisons test.

Increasing amplitude of depolarizing current steps led a significant increase in action potential firing rate in D2 SPNs (F_11,297_ = 101.6, p < 0.0001), there was a main effect of genotype (F_2,27_ = 5.128, p = 0.0129), and there was a significant interaction between genotype and current injection (F_22,297_ = 3.152, p = < 0.0001, Fig. 5a,b). The source of this difference was decreased D2 SPN excitability in the MMP-1OE and MMP-1OE/PAR-1KO mice compared to control mice between 40 pA injections (p_MMP-1OE_ = < 0.0001, p_MMP-1OE/PAR-1KO_ = 0.0085) and 80 pA injections (p_MMP-1OE_ = 0.0013, p_MMP-1OE/PAR-1KO_ = 0.0379).

Dendritic complexity of D2 SPNs differed as a function of genotype (Fig. 5c,d,e). Control D2 SPNs had decreased complexity between 40 μm (p = 0.0263) and 70 μm (p = 0.0287) compared to MMP-1OE mice. Furthermore, MMP-1OE/PAR-1KO mice displayed increased complexity compared to control mice between 80 μm (p = 0.0259) to 100 μm (p = 0.0006). At 110 μm, D2 SPNs in MMP-1OE/PAR-1KO mice displayed increased complexity compared to control (p = 0.0039) and MMP-1OE mice (p = 0.0329). As expected there was increased complexity as a function of distance from the soma (F_25,800_ = 143.5, p < 0.0001). There was no main effect of genotype (F_2,32_ = 2.59, p = 0.0.0906), but there was a significant interaction between these variables (F_50,800_ = 1.972, p = 0.0001). Additionally, there were fewer tertiary branches on D2 SPNs of control mice compared to MMP-1OE/PAR-1KO mice (p = 0.0369, Fig. 5f). There was a main effect of branching on number of branches (F_2,64_ = 30.26, p < 0.0001), but no main effect of genotype on number of branch type (F_2,32_ = 1.962, p = 0.1571) or an interaction between genotype and branch complexity (F_4,64_ = 1.384, p = 0.2496). Branch length did not differ across the three genotypes (Fig. 5g). There was an effect of branching on length (F_2,64_ = 10.01, p = 0.0002), but no effect of genotype (F_2,32_ = 1.617, p = 0.2143), or an interaction of these variables (F_4,64_ = 1.216, p = 0.3128).

### Alterations in amphetamine induced locomotion

**Figure 6:**
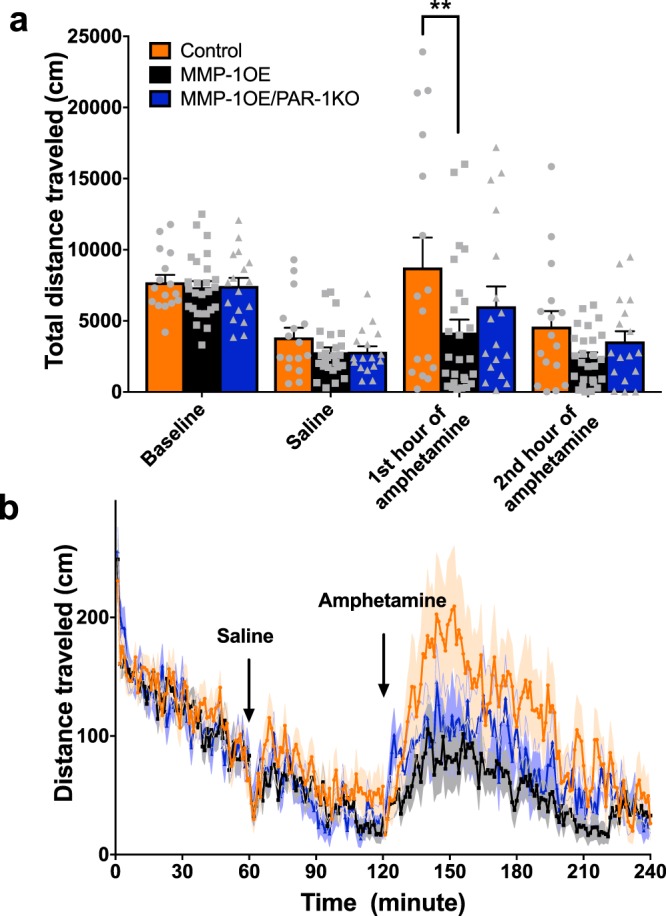
Disruption of amphetamine induced locomotion in MMP-1OE mice. (**a**) A timeline of ambulatory distance for the mice. Testing occurred as follows: one-hour baseline, one hour post saline injection, two hours post amphetamine injection. (**b**) Adult (P50–70) MMP-1OE mice exhibit a blunted locomotive response to amphetamine injection in comparison to age matched controls (p = 0.0005). Spontaneous locomotion and response to saline are unaltered. There was a main effect of amphetamine injection (F_3,168_ = 26.52, p < 0.0001), but no main effect of genotype (F_2,56_ = 3.036, p = 0.0560). The drug by genotype interaction was approaching statistical significance therefore we preformed post hoc tests (F_6,168_ = 1.794, p = 0.1030).

Motor behavior was assessed via evaluation of both spontaneous and amphetamine induced locomotion in control, MMP-1OE, and MMP-1OE/PAR-1KO mice (Fig. 6a,b). There were no differences between spontaneous locomotion and locomotor response to saline as a function of genotype. Amphetamine injection increased locomotor activity [total distance traveled] in all three genotypes. There was a main effect of amphetamine injection (F_3,168_ = 26.52, p < 0.0001), but no main effect of genotype (F_2,56_ = 3.036, p = 0.0560). The drug by genotype interaction approached statistical significance (F_6,168_ = 1.794, p = 0.1030). *A priori* we hypothesized that MMP1-OE mice would display altered response to amphetamine challenge, therefore we evaluated the response across genotypes during the amphetamine-exposed period. The effect of amphetamine was decreased in MMP-1OE mice compared to control mice (p = 0.0005).

## Discussion

Here, we report that genetic over-expression of MMP-1 triggers increased synaptic inhibition and excitation onto D1 SPNs, but not D2 SPNs in the nucleus accumbens core. Moreover, we show that inhibition and excitation are normal in MMP-1 overexpressing mice that lack PAR-1, a known substrate expressed on neurons and glia^5,7^. In addition, increased inhibitory and excitatory synaptic input onto D1 SPNs was associated with increased dendritic complexity of D1 SPNs and a blunted locomotive response to amphetamine. A subset of morphological and behavioral effects was absent in MMP1-OE/PAR-1KO mice. Taken together, these results suggest a significant role for the MMP-1/PAR-1 axis in the regulation of striatal physiology and behavioral functions.

There are several possible mechanisms that may account for the increased PSC frequency we observed in D1 SPNs from MMP-1OE mice. Non-mutually exclusive possibilities include: altered excitability of the presynaptic cell, altered vesicular release, or altered dendritic complexity. Though increased excitability of presynaptic cells may have contributed to the increase in sIPSC frequency, this is unlikely to be the only mechanism involved in that we observed increases in both sIPSC frequency and action potential-independent mIPSC frequency. Increased presynaptic release probability is also a plausible contributor to the increase in PSC frequency. Further studies are needed to identify which presynaptic inhibitory inputs onto D1 SPNs may be affected by MMP-1OE. The most probable source of increased inhibitory synaptic input to SPNs are striatal interneurons. GABAergic interneurons, although a minority of cells in the striatum, are potent sources of inhibitory synaptic input to SPNs. Multiple classes of interneurons make both somatic and dendritic connections onto both D1 and D2 SPNs^30–35^. By contrast, collateral inhibition from other SPNs seems less plausible for several reasons. First, excitability of both D1 and D2 SPNs was decreased, a profile that would not be consistent with increased release. Second, while D1 SPNs preferentially synapse onto D1 SPNs^36^, D2 SPNs synapse onto both D1 and D2 SPNs. Given that we detected increased IPSC frequency only in D1 neurons, a global increase in presynaptic activity of D2 SPNs would be expected to impact both populations. It is worth noting that although the microcircuitry of the ventral striatum might be similar to that of the dorsal striatum, it has yet to be fully elucidated.

Increased dendritic complexity can also lead to an increase of IPSC and mEPSC frequency because it allows for increased synaptic connectivity^37,38^. Whole-cell recordings with a potassium chloride-based internal solution can accurately detect currents originating in dendrites up to 100 μm from the soma^39^. The increased dendritic complexity seen in D1 SPNs of the MMP-1OE mice occurred between 60 μm and 80 μm from the soma, thus, the increased synaptic transmission observed may be due in part to the altered dendritic tree. Although we observed increased dendritic complexity in D2 SPNs of MMP-1OE and MMP-1OE/PAR-1KO mice, similar alterations in PSC frequency were not observed. This dissociation may be due to the increased dendritic complexity occurring distal to the soma, likely beyond the spatial detection limit of PSCs.

Both PAR-1 dependent and PAR-1 independent effects of MMP1-OE were also evident in the passive and active membrane properties of SPNs: D1 and D2 SPN excitability was decreased in the MMP-1OE mice compared to controls. These effects may be due in part to increased dendritic complexity^40^. The excitability and complexity of D1 SPNs was normalized in the MMP-1OE/PAR-1KO mouse, but the decreased excitability and complexity of D2 SPNs was not. Interestingly we observed an increase in the membrane time constant and resistance of D1 SPNs of MMP-1OE/PAR-1KO mice; this may have resulted in no net change in excitability in these mice, these changes in membrane properties likely have opposing effects. Assessing the properties and expression of potassium channels (e.g., those mediating the inward rectifying potassium current), as they are key regulators of SPN excitability, will help to further elucidate the mechanisms at play.

The effects of MMP-1OE on synaptic transmission were selective to D1 SPNs. Neither IPSC or EPSC frequency differed as a function of genotype in D2 SPNs. Published work using single cell RNAseq revealed PAR-1 expression in astrocytes, D1 SPNs, and D2 SPNs in the striatum, with the greatest expression in astrocytes^41^. PAR-1’s actions could thus be through neuronal or astrocytic mechanisms. With respect to the latter possibility, PAR-1 has been implicated in astrocyte-neuron interactions, also known as the tetrapartite synaptic model^42^ Moreover, in the striatum, SPNs activate subsets of astrocytes, which in turn activate homotypic SPNs^43^. PAR-1 signaling in a D1 SPN – astrocyte microcircuit, a tetrapartite synapse, could thus also contribute to the effects we detected. A neuronal PAR-1 mechanism at simple pre-synaptic/post-synaptic neuron model, may also contribute to the phenotype observed^10^. Studies will need to probe the cell specific and subcellular localization of PAR-1 to further understand the alterations observed. Furthermore, activation of PAR-1 releases an N-terminal domain which acts on endothelial cells and inhibits angiogensis^44^. The role of the released peptide in altering synaptic transmission in the brain has yet to be examined. Another possibility is unidentified endogenous peptide ligands that can substitute for the cleavage generated tethered ligand may exist in the brain. Acute PAR-1 activation alters glutamate uptake and thus excitatory transmission in the hippocampus^45^. Whether a similar mechanism occurs at inhibitory synapses in the striatum due to chronic PAR-1 activation has yet to be determined.

In addition to altered synaptic function of D1 SPNs, we found increased dendritic complexity in both D1 and D2 SPNs of MMP-1OE mice. The increased dendritic complexity seen in the D1 SPNs of MMP-1OE mice is not observed in the MMP-1/PAR-1KO. Consistent with this, prior studies have shown that PAR-1 activation regulates calcium flux in neurons and increases hippocampal dendritic complexity^6,46^. Given that the increased complexity seen in the D1 SPNs was normalized in the MMP-1OE/PAR-1KO mouse, this phenotype is likely PAR-1 dependent. By contrast, D2 dendritic arbor was not normalized in MMP-1OE/PAR-1KO mice, suggesting that PAR-1 independent effects may be driving these changes. Possibilities include breakdown of the extracellular matrix (ECM) which has been linked to dendritic growth and altered synaptic transmission^47^. Moreover, disruption of the ECM is found in many neurodegenerative states, such as Alzheimer’s disease, epilepsy, and multiple sclerosis^48^.

Although the dorsal striatum is a major regulator of locomotor behavior, recent evidence has implicated the ventral striatum as a player as well. For example, loss of dopamine D2 signaling in the ventral striatum can lead to a blunted locomotor response^28^. Similarly, in a mouse model of autism, decreased inhibitory transmission onto D1 SPNs in the nucleus accumbens was correlated with increased spontaneous locomotion^49^. Our results reveal a blunted locomotive response to amphetamine in MMP-1OE mice. The phenotype we observed may be due to the altered excitability of D1 and D2 SPNs or the synaptic inputs onto these neurons. While we focused on the ventral striatum, the transgenic approach we used was global. Thus, we cannot rule out a contribution of other brain regions or mechanisms to this motor phenotype. Potential contributors include altered dorsal striatal function, altered presynaptic dopamine neuron function or post-synaptic dopamine receptor signaling. Spontaneous locomotion was not altered in these mice suggesting that the two motor behaviors have distinct neuronal mechanisms. These studies highlight the complex underpinning of distinct motor functions and its neurological basis.

A caveat of our present studies is that we were unable to examine mice that lack PAR-1 in the absence of MMP-1 overexpression. The limited litter sizes and high *in utero* mortality reported for PAR-1KO mice made this experiment practically unattainable^50^. Interestingly, crossing the MMP-1OE mouse to the PAR-1KO mouse rescued the early mortality previously reported in this strain, enabling the experiments we performed. In light of this caveat, we note that gross brain structure is normal in PAR-1 knockout mice^20^. As for the MMP-1OE/PAR-1KO mice, although the murine orthologue of MMP-1 is not expressed in the absence of pathology^46^, additional PAR-1 activators including MMP-3 and -13 are physiologically expressed^9,21^. Importantly, while the increased synaptic transmission observed in the MMP-1OE mice were not observed in the MMP-1OE/PAR-1KO, a portion of the changes (e.g., intrinsic excitability, dendritic arborization in D2 SPNs) persisted in the MMP-1OE/PAR-1KO mouse. This suggests that MMP-1 may be acting via a combination of PAR-1 dependent and independent mechanisms, consistent with the fact that MMP-1’s actions are likely mediated by multiple mechanisms including its potential to generate integrin binding ligands^51^. Our work was performed on mice that overexpressed MMP-1 or lacked PAR-1 throughout development, which may bear relevance to MMP-1 promoter polymorphisms that affect expression in a relatively persistent manner. Taking into considerations the role of the MMP-1/PAR-1 axis inflammatory and ischemic pathology, it is of importance to evaluate the effect of persistent PAR-1 activation on physiology^19^. However, we acknowledge that disease states including PD may instead involve a temporally restricted upregulation of this product by activated glia, and thus the pattern of changes observed may differ from long-term upregulation^52^. These data provide an initial evaluation of MMP-1/PAR-1 signaling in the striatum. Further studies will need to examine the effect of more temporally and spatially restricted signaling such administration of recombinant MMP-1 into the striatum.

MMPs play a vital role in facilitating excitatory synaptic plasticity in the hippocampus that is specific for both distinct MMPs and lamina^21^. Our results revealed a specificity of MMP-1OE for D1 SPNs in the nucleus accumbens core which is correlated with increased dendritic complexity. The altered motor behavior is present in both mouse models suggesting MMP-1’s actions on locomotion may not be PAR-1 mediated. These data are of importance because of the alteration of both MMP-1 and PAR-1 in several neurological diseases with a motor dysfunction component^17,18^. The MMP-1/PAR-1 axis may contribute the locomotor phenotype observed in these diseased states. This opens the door for treatments focusing on MMP-1 and/or PAR-1.

## Materials and Methods

### Animals

Bacterial artificial chromosome (BAC) D2 enhanced green fluorescent protein (EGFP) and BAC D1 tdTomato mice were crossed to obtain a mouse that expresses both D2-EGFP and D1-tdTomato^53,54^. The MMP-1 over expressing transgenic (MMP-1OE) mice are global overexpressers, displaying no aggregates, and have been previously validated^46^. The human MMP-1 (hMMP-1) cDNA (Dr. J D’Amriento, Columbia University) was subcloned downstream of the GFAP promotor. The hMMP-1, is an orthologue of the mouse MMP-1a and more importantly is an activator of the mouse PAR-1^19,55^. The MMP-1OE mice were crossed with the global PAR-1 knock out mice, which have also been validated (MMP-1OE/PAR-1KO, F2r^tm1Ajc^Jackson Laboratory). All mice were maintained on the C57BL/6 background. Mice were group-housed in barrier cages in rooms with a 12-hour:12-hour light/dark cycle and permitted free access to food and water. The procedures performed were in accordance with and approval by Georgetown University Animal Care and Use Committee (GUACUC).

Both male and female mice were used for all studies and were combined when found to not be statistically different. We used two different mating pair strategies. The first strategy resulted in wild-type control and MMP-1OE mice. These mice were used for whole-cell recordings, morphological reconstruction, and the locomotion assay. The second strategy produced all three genotypes: control, MMP-1OE and MMP-1OE/PAR-1KO mice. This strategy generated smaller numbers of control and MMP-1OE, therefore, for the electrophysiology and morphological reconstruction experiments we used MMP-1OE/PAR-1KO mice from the second mating strategy and compared them to control and MMP-1OE from the first mating strategy. When a litter contained all three genotypes from the second breeding strategy these mice were used for the locomotion assay; we were able to obtain 2 control, 8 MMP-1OE and 17 MMP-1OE/PAR-1KO mice for locomotor testing across several litters from the second breeding strategy. Because control and MMP-1OE animals from this second breeding strategy did not differ significantly from the first breeding strategy, data were combined for further analysis. The control data set (wild-type animals) generated for this study were used in a concurrent, but separate set of statistical analyses. These analyses, comparing the properties of D1 and D2 neurons in wild-type animals were published^29^. The present manuscript, while using the same control data set, does not duplicate any of the statistical analyses previously reported.

The litter size of PAR-1KO mice (4 pups/litter) is significantly smaller than that of control (7 pups/litter, p = 0.0097), MMP-1OE (7 pups, p = 0.0097), and MMP-1OE/PAR-1KO mice (6 pups/litter, p = 0.0238). Due to this constraint, the studies focused on control, MMP-1OE and MMP-1OE/PAR-1KO mice. Interestingly, crossing the MMP-1OE mouse to the PAR-1KO mouse rescued this phenotype.

### Brain slice preparation

Slices were prepared from postnatal day 17–23 (P17–23) mice^56^. Mice were sacrificed by decapitation in agreement with the guidelines of the American Veterinary Medical Association Panel on Euthanasia and the GUACUC. The whole brain was removed and placed in an ice-cold cutting solution containing (in mM): NaCl (87.3), KCl (2.7), CaCl_2_ (0.5), MgSO_4_ (non-hydrate) (6.6), NaH2PO_4_ (1.4), NaHCO_3_ (26.0), dextrose (25.0), sucrose (75.1). A Vibratome 3000 Plus was used to prepare 250 μm thick striatal coronal slices. The slices were incubated in artificial cerebrospinal fluid (aCSF) containing (in mM): NaCl (123.9), KCl (4.5), Na_2_HPO_4_ (1.2), NaHCO_3_ (26.0), CaCl_2_ (2.0), MgCl_2_ (1.0), and dextrose (10.0) at 305 mOsm at 32 °C for 30 minutes. The slices were incubated for an additional 30 minutes in the same solution at room temperature. Solutions were continuously bubbled with 95% O_2_/5% CO_2_ to maintain a pH of 7.4.

### Recording

Slices were visualized using an upright microscope (E600FN, Nikon) equipped with Nomarski optics and a 60X water immersion objective with a long working distance (2 mm) and high numerical aperture (1.0). Recording electrodes with a resistance of 4–6 MΩ were prepared from borosilicate glass capillaries.

A KCl-based internal solution containing (in mM): KCl (145.0), HEPES (10.0), ATP-Mg (5.0), GTP-Na (0.2), EGTA (5.0) and adjusted to pH 7.2 with KOH was used for all recordings. Voltage-clamp recordings were achieved using the whole-cell configuration method at a holding voltage of -70 mV using the MultiClamp 700B amplifier. All recordings were performed at room temperature, 22–24 °C. Recordings were performed from D1 and D2 SPNs in the area directly surrounding the anterior commissure (nucleus accumbens core). Cell type was determined by fluorescence expression (red only or green only) and firing pattern. Responses to increasing hyperpolarizing and depolarizing current injections (20 pA steps) were obtained to assess passive properties, and action potential firing pattern and number. Access resistance was monitored periodically during the experiment and recordings with a >20% change were discarded. Recordings were filtered at 2 kHz with a low-pass Bessel filter and digitized at 20 kHz using a personal computer equipped with Digidata 1440 data acquisition board and pCLAMP10 software.

Working solutions of tetrodotoxin (TTX, 1 μM) and bicuculline methobromide (BMR, 25 μM) were prepared in aCSF and locally applied to the slice via Y tube^57^. Prior to drug application, the whole cell currents were acquired for five minutes to obtain spontaneous inhibitory postsynaptic currents (sIPSCs), at which time TTX was applied to study miniature IPSCs (mIPSCs), lastly BMR was applied to evaluate GABA_A_ mediated tonic current and miniature excitatory PSCs. NBQX was not used for the measurement of IPSCs as to not disturb the network activity^58^. The rapid decay kinetics of AMPA-mediated EPSCs allowed us to exclude them from IPSC analysis^59–62^. Moreover, EPSC contamination for our recordings is minimal as seen by the low frequency of mESPCs. As in acute corticostriatal slices, sEPSC and mEPSC frequency are equivalent, we only recorded mEPSCs^60^.

### Morphological Reconstruction

Whole-cell recordings were obtained using the internal solution detailed above with the addition of 0.5% biocytin. Neurons were then injected with 35 steps of hyperpolarizing and depolarizing current injections in current clamp mode (100 ms, 20 pA). After approximately 15 minutes of recording, an outside-out seal was obtained to prevent leakage after filling, and slices were allowed to rest for another 45 minutes before fixation. Slices were then fixed with 4% sucrose/4% paraformaldehyde in 0.1 M phosphate buffered saline (PBS) at room temperature for 2 hours. The slices were subsequently washed with 0.5% triton-X in 1X PBS for at least 30 minutes. Slices were then incubated in avidin-fluorescein (2.5 ul/mL) for 2 hours. Afterwards, slices were washed overnight and then mounted with Vectashield, H-1000 mounting medium to be imaged.

Imaging of slices was performed using a ThorLabs resonance laser scanning confocal microscope with 488 nm and 547 nm argon laser on a Nikon Eclipse FN1 upright microscope with a 60x water immersion lens (1.0 N.A.) or a 40X lens (0.9 N.A.) or a 20x lens (0.5 N.A.).

### Locomotion assay

A week prior to behavioral testing, animals were handled to minimize confounding results due to stress. Mice were placed in an acrylic locomotor arena (40 × 40 × 30 cm, l x w x h) and allowed to freely explore for 1 hour. After 1 hour, mice were injected with saline 0.1 ml/10 g body weight, intraperitoneal (ip) and allowed to freely explore in the area. After an hour, animals were injected with d-amphetamine sulfate (2.5 mg/kg, ip) and locomotor activity was monitored for an additional 2 hours^63,64^. Total distance for each hour block was analyzed. While not specific for striatal function, the prolocomotor effects of amphetamine are primarily driven by dopamine release in the striatum accordingly dopamine antagonists^65^ in the nucleus accumbens block d-amphetamine induced locomotor activity^66^.

### Experimental design and Statistical analysis

Bar graphs of frequency, peak amplitude, rise time, and decay time displayed average values and standard error of the mean. Data were analyzed by cell number and only 1–2 cells were recorded per animal. Outliers were determined using the ROUT test with a stringent exclusion threshold (Q = 1%) and were excluded from all datasets as follows: passive properties (MMP-1OE D1 SPNs = 1 cell/1 animal), active properties (Control D2 SPNS = 1 cell/1 animal, MMP-1OE/PAR-1KO D2 SPNs = 1 cell/1 animal), current data (Control D1 SPNs = 1 cell/1 animal, MMP-1OE D1 SPNs = 2 cells/2 animals, MMP-1OE/PAR-1KO = 2 cells/2 animals, control D2 SPNs = 2 cells/2 animals), dendritic architecture (Control D1 SPNs = 3 cells/3 animals, MMP-1OE D1 SPNs = 2 cells/2 animals, MMP-1OE/PAR-1KO D1 SPNs = 1 cell/1 animal, Control D2 SPNs = 2 cells/2 animals, MMP-1OE D2 SPNs = 4 cells/4 animals, MMP-1OE/PAR-1KO D2 SPNs = 2 cells/2 animals) and behavior (control = 1, MMP-1OE = 4, MMP-1OE/PAR-1KO = 1). For the dendritic architecture data, the Sholl results were used. A cell was considered excluded if it contained 3 or more points that were found to be outliers. The N reported only includes the data used for statistical analysis.

IPSCs/EPSCs were measured using ClampFit template search and visually confirmed^60,61^. Frequency of PSCs was measured directly as the number of events divided by the length of the recording. Resting membrane potential was measured at I = 0. Passive properties were measured from the voltage response to hyperpolarizing current injections. Action potential firing rate was measuring manually from depolarizing current injections. Bioyctin injected cells were traced using the Fiji NeuronJ plugin and dendritic arborization was analyzed using the Sholl analysis plugin^67^.

Electrophysiological and morphological reconstruction data were analyzed separately for the D1 and D2 populations as a function of genotype. The data were tested for normality and the appropriate parametric or non-parametric tests were used. For IPSC/EPSC parameters and passive properties statistical significance was assessed using the non-parametric Kruskal-Wallis test with Dunn’s multiple comparison test. Neuronal firing pattern was analyzed by 2-way ANOVA with genotype as a between subject factor and current intensity as a within subject factor. Morphological analyses were conducted using a 2-way ANOVA for the Sholl analysis, Kruskal-Wallis for the length and individual branch count parameters. Locomotion assay data were analyzed by 2-way ANOVA with genotype as a between subject factor and time as a repeated measure; data summed across hourly blocks were analyzed via Kruskal Wallis test. In all cases *P* values less than 0.05 were considered to be statistically significant.

## Data Availability

The datasets generated during and/or analyzed during the current study are available from the corresponding author on reasonable request.
